# Short-term outcomes and cost-effectiveness of laparoscopic gastrectomy with articulating instruments for gastric cancer compared with the robotic approach

**DOI:** 10.1038/s41598-023-36601-7

**Published:** 2023-06-08

**Authors:** Chang Min Lee, Sungsoo Park, Sung Hyun Park, Ki-Yoon Kim, Minah Cho, Yoo Min Kim, Woo Jin Hyung, Hyoung-Il Kim

**Affiliations:** 1 Department of Surgery, Korea University College of Medicine, Seoul, South Korea; 2 Department of Surgery, Yonsei University College of Medicine, 50 Yonsei-ro, Seodaemun-gu, 120-752 Seoul, South Korea

**Keywords:** Gastrointestinal cancer, Stomach

## Abstract

To overcome the limitations of laparoscopic surgery, robotic systems have been commonly used in the era of minimally invasive surgery despite their high cost. However, the articulation of instruments can be achieved without a robotic system at lower cost using articulating laparoscopic instruments (ALIs). Between May 2021 and May 2022, perioperative outcomes following laparoscopic gastrectomy using ALIs versus robotic gastrectomy were compared. A total of 88 patients underwent laparoscopic gastrectomy using ALIs, while 96 underwent robotic gastrectomy. Baseline characteristics were similar between the groups except for a higher proportion of patients with a medical history in the ALI group (*p* = 0.013). Clinicopathologic and perioperative outcomes were not significantly different between the groups. However, the operation time was significantly shorter in the ALI group (*p* = 0.026). No deaths occurred in either group. In conclusion, laparoscopic gastrectomy using ALIs was associated with comparable perioperative surgical outcomes and a shorter operation time compared to robotic gastrectomy in this prospective cohort study.

## Introduction

Minimally invasive surgery (MIS) has been increasingly utilized because of its beneficial effects on postoperative pain, functional recovery, and hospital stay, compared to open surgery^1–4^. MIS is also now considered a standard alternative option for the surgical treatment of gastric cancer^5^, with recent multicenter prospective randomized clinical trials showing that the oncologic safety of laparoscopic gastrectomy is not inferior to that of open surgery^6–8^. Despite the good results achieved to date, surgeons continue to develop new techniques and instruments to enhance the surgical quality of MIS.

One major innovation in the field of MIS has been the introduction of robotic surgical systems. Since its introduction in 1988^9^, the da Vinci^®^ Surgical System (Intuitive Surgical, Sunnyvale, CA, USA) has been used to overcome the drawbacks of conventional laparoscopic surgery and has become a feasible option for MIS in patients with gastric cancer^10,11^. Surgeons have been fascinated by the potential advantages of this system, including better field of view, articulated movements of surgical instruments, and the presence of a surgical console^12–14^. In particular, the articulation provided by the robotic system has solved many of the issues with earlier laparoscopic gastrectomy.

Although evidence is accumulating on the clinical outcomes of robotic gastrectomy for patients with gastric cancer, several issues remain regarding the use of robotic surgical systems. A recent multicenter prospective trial comparing short-term outcomes between robotic and laparoscopic gastrectomy demonstrated significantly longer operation times and higher costs with robotic surgery^12^. In general, the docking time and other instrument-preparation times (e.g., exchanging the instrument, switching the arms) contribute to the longer operation time of robotic gastrectomy compared to laparoscopic surgery^15,16^. These time-consuming activities reduce the benefits of articulating instruments because articulating functionality is not always necessary during gastrectomy procedures. In many fields of surgery, a “hybrid” approach is used, where both laparoscopic and robotic procedures are selectively performed to complete an operation^17–20^. This suggests that the robotic surgical system may be useful in only a selected proportion of gastrectomies. Therefore, it is natural to consider using articulating instruments only when necessary, resulting in strategic cost reduction, an important consideration because of the high costs associated with robotic surgery.

Over the years, articulating laparoscopic instruments (ALIs) have been introduced in several fields of MIS^21–23^. While conventional laparoscopic instruments (CLIs) are stiff, ALIs have multiple degrees of freedom (DOF). To assess the usefulness of ALIs, compared to robotic surgery, we developed a prospective database of patients undergoing MIS for gastrectomy. In this study, we compared the clinical outcomes between robotic gastrectomy and laparoscopic gastrectomy using ALIs in patients who underwent surgery over a 1-year period.

## Materials and methods

### Study design

This prospective cohort study was approved by the Institutional Review Board of Severance Hospital, Yonsei University Health System (4-2021-0409). All the surgeries were done by a single surgeon. The study protocol was registered at ClinicalTrials.gov (NCT04972149). The primary endpoint was to examine the proportion of major complications. The secondary endpoints were operation time, amount of bleeding, and hospital stay. All methods are in accordance with relevant guidelines and regulations.

### Participants

**Figure 1:**
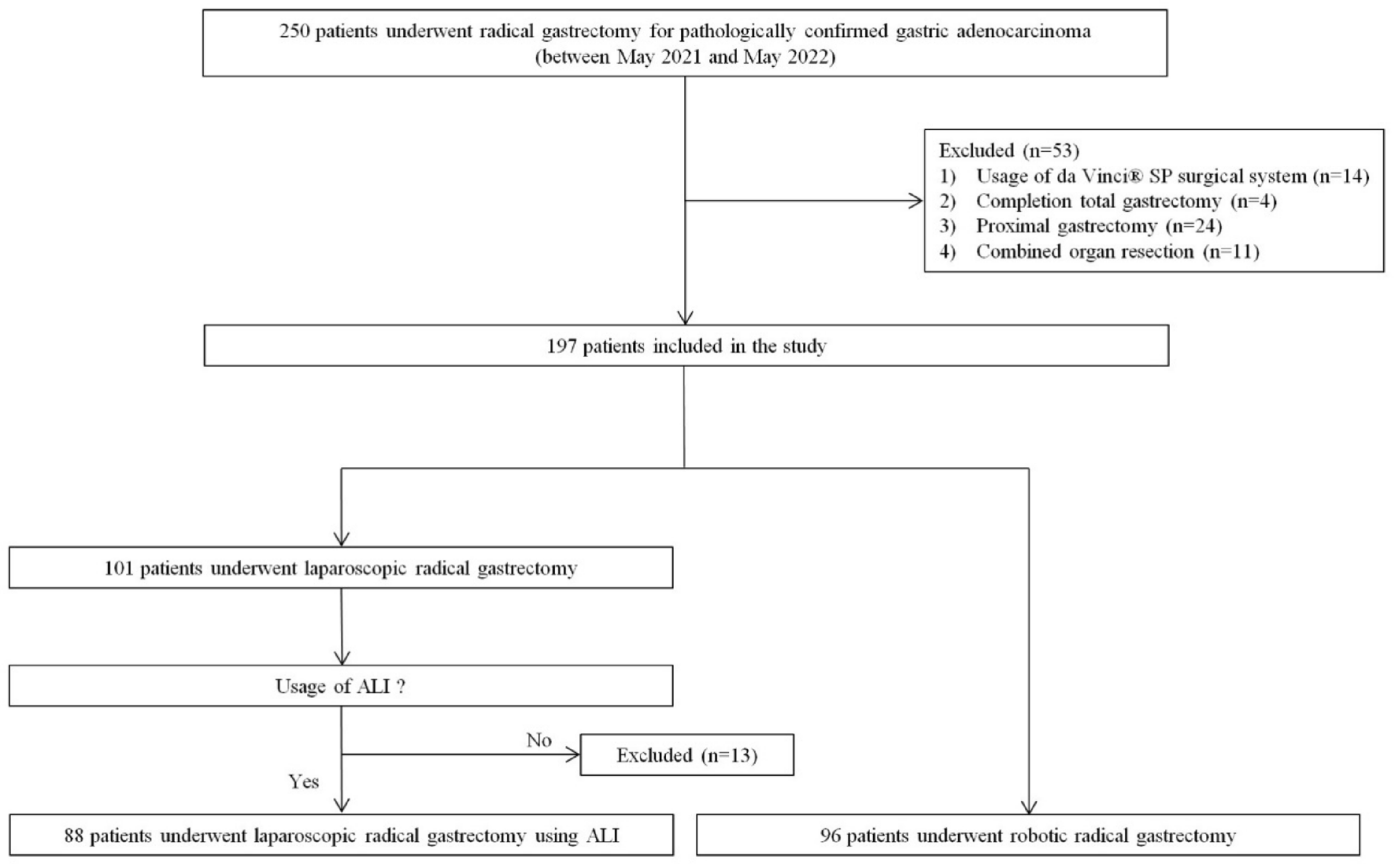
Patient flow diagram. Of the 250 patients who underwent gastrectomy for gastric cancer during the 1-year study period, 66 patients met the exclusion criteria and were therefore excluded from the study. The remaining 184 patients included 88 patients who underwent laparoscopic surgery using ALIs and 96 who underwent robotic surgery. ALI, articulating laparoscopic instrument.

The data were prospectively collected for 1 year between May 2021 and May 2022. ALI was used in all laparoscopic gastrectomies performed during the study period. All the patients undergoing minimally invasive gastrectomy were asked to participate in the study. A total of 250 patients were enrolled in this study. ALIs were used in all laparoscopic gastrectomies performed during the study period. The indication for robot or laparoscopic gastrectomy was the same. The patient chose the approach method after receiving a full explanation of the cost and potential benefits of the robot gastrectomy. Informed consent for radical gastrectomy was obtained from all patients. Inclusion criteria were as follows: patients who consented to undergo surgery using ALIs or the robotic surgical system. Patients were excluded if they underwent any of the following: (1) robotic surgery using the da Vinci^®^ SP Surgical System (Intuitive Surgical), (2) completion of a total gastrectomy for remnant gastric cancer, (3) proximal gastrectomy, or (4) combined organ resection for T4b gastric cancer, double primary malignancies, or other diseases. The final analyses included 88 patients who underwent laparoscopic surgery using ALIs (“ALI group”) and 96 patients who underwent robotic surgery (“robotic group”) (Fig. 1).

### Instruments

**Figure 2:**
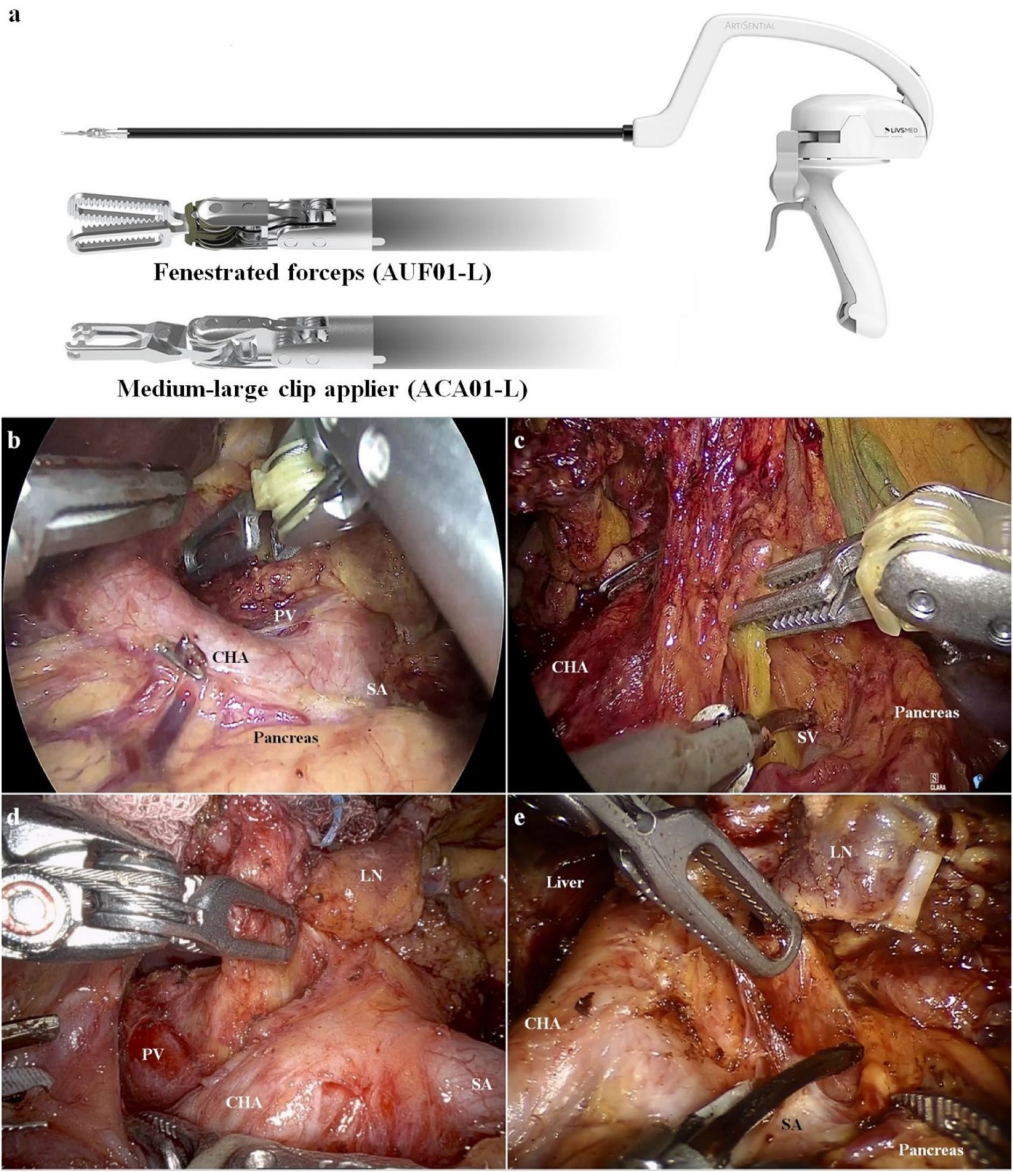
Articulating function for supra-pancreatic lymph node dissection. (**a**) Overview of the fenestrated forceps and medium-large clip applier. (**b**) Snapshot of the 12a area lymph node dissection using ALI. (**c**) Lifting lymph nodes without disturbing the movement of the energy device is necessary. Dissection of the 11p area also requires upward traction of the lymph nodes creating space for the approaching energy devices. The same applies to robots adequately exposing the deep-seated 12a (**d**) and 11p (**e**) tissues. CHA, common hepatic artery; PV, portal vein; SA, splenic artery; SV, splenic vein; LN, lymph node.

In the ALI group, ArtiSential fenestrated forceps, and a medium-large clip applier (AUF01-L and ACA01-L, LivsMed, Seongnam, Korea) were used during the lymphadenectomy. The mechanical and functional characteristics have been previously described^24^. Briefly, the fenestrated forceps and clip applier have wrist functions that can reach the target tissue at an appropriate angle regardless of the location of the ALIs. Both instruments exhibit similar characteristics to the robotic arm. They require an 8-mm-sized trocar and have a similar degree of freedom and angle. The tip of the instruments resembles those of robots (Fig. 2a). Five ports were placed for the laparoscopic approach, including the umbilicus for the scope as previously described^25^. The port placed in the right upper part of the abdomen (8-mm port for the grasper and fenestrated forceps) and right lower part of the abdomen (12-mm port for the energy device and clip applier) were used for the operator’s left and right hands. Two additional ports were placed in the left upper and left lower parts of the abdomen for the assistant. In the robotic group, the da Vinci^®^ Xi Surgical System (Intuitive Surgical) was used during the lymphadenectomy as previously described^26^.

### Surgical procedures

After entering the operating room, the patient was placed supine on the operating table, and general anesthesia was initiated. The operating table was then adjusted to place the patient in a reverse Trendelenburg position.

The falciform ligament and left lobe of the liver were pulled in a cephalad direction by combined suture retraction^27^. Lymphadenectomy for curative gastrectomy was performed based on the Korean Gastric Cancer Association guidelines and the Japanese Gastric Cancer Treatment Guidelines 2014 (ver. 4)^28,29^. ALIs (Fig. 2b,c) or a robotic surgical system (Fig. 2d,e) were used to facilitate D1+ or D2 lymphadenectomy in all patients. After completion of the lymphadenectomy, Billroth I or II or Roux-en-Y anastomosis were performed to ensure gastrointestinal tract continuity.

### Postoperative management

Postoperative management of both patient groups followed the same protocol. All patients received intravenous patient-controlled analgesia with fentanyl for the first 2 days after surgery. On postoperative day 3, the analgesic regimen was converted to oral tramadol plus acetaminophen (Ultracet ER, Janssen-Ortho, LLC, Gurabo, Puerto Rico). Sips of water and a clear liquid diet were resumed in the morning and afternoon of postoperative day 1, respectively. A soft diet was permitted in the afternoon of the second day after surgery. Depending on recovery status, patients were discharged the third day after surgery or later^30^.

Patients returned for follow-up visits at 1 month postoperatively, every 3 months for 1 year, and then every 6 or 12 months thereafter, depending on their disease status during the follow-up period.

### Data collection and outcome assessment

The following patient characteristics and outcome data were recorded: age, sex, body mass index, American Society of Anesthesiologists physical status class, previous surgical or medical history (hypertension, diabetes mellitus, coronary heart disease, respiratory illness, and other comorbidities associated with the major organs), extent of lymphadenectomy, extent of gastric resection, type of reconstruction method, operation time (defined as the time from skin incision to skin closure), volume of intraoperative blood loss, conversion to laparoscopic or open surgery, number of retrieved lymph nodes (LNs), T and N classification, number of days until passing gas postoperatively, and length of hospital stay. Perioperative (within the first 30 days after surgery) complications and mortality were also recorded, with complications categorized according to the Clavien–Dindo (C–D) classification system^31^. Patient characteristics, clinicopathologic outcomes, and postoperative outcomes were compared between the ALI and robotic groups.

### Statistical analysis

Statistical analyses were performed using SPSS version 25.0 (SPSS, Inc., Chicago, IL, USA). We compared the patient characteristics and clinicopathologic outcomes between the groups using the Pearson chi-square test or Fisher’s exact test for categorical variables and the independent t-test or Mann–Whitney test for continuous variables. Mean values are presented with standard error throughout the manuscript. Any p-value < 0.05 was considered statistically significant.

## Results

A total of 184 patients were included in the final analysis: 88 in the ALI group and 96 in the robotic group. Baseline patient characteristics were similar between the two groups, except for the presence of medical history (Table 1). Significantly more patients had a medical history in the ALI group compared to the robotic group (67% vs. 49%, *p* = 0.013).

**Table 1:** Baseline characteristics of patients who underwent gastrectomy for gastric cancer using either laparoscopic articulating instruments (ALI group) or a robotic surgical system (robotic group) for lymph node dissection.

Variables	Group		*p*
	ALI (n = 88)	Robotic (n = 96)	
Age at operation, y	64.3 ± 10.5	61.8 ± 12.7	0.156
Sex			0.906
Male	53 (60.2)	57 (59.4)	
Female	35 (39.8)	39 (40.6)	
Body mass index, kg/m^2^	23.9 ± 2.9	24.0 ± 3.2	0.860
Surgical history			0.564
None	47	48	
ESD or stomach	4	2	
Abdominal	22	23	
Extra-abdominal	15	23	
ASA physical status class			0.116
1	0	5	
2	52	57	
3	35	34	
4	1	0	
Medical history^a^			0.013
No	29 (33.0)	49 (51.0)	
Yes	59 (67.0)	47 (49.0)	

Data are expressed as mean ± standard error or number (percentage).

ALI, articulating laparoscopic instrument; ASA, American Society of Anesthesiologists; ESD, endoscopic submucosal dissection.

^a^Hypertension, diabetes mellitus, coronary heart disease, respiratory illness, and other comorbidities associated with major organs.

Of the operative outcomes, there were no significant differences between the two groups except for operation time (Table 2). The operation time was significantly longer in the robotic group versus the ALI group (186.5 ± 58.6 min vs. 168.6 ± 48.9 min, *p* = 0.026). Postoperative recovery outcomes and complications did not differ between the ALI and robotic groups (Table 3). There were no perioperative deaths or C-D class IV complications in either group. The total cost was higher in the robotic group compared to the ALI group (11,178 ± 202 USD vs. 9822 ± 162 USD, *p* < 0.001). The difference was caused by the operation and hospital stay costs (*p* < 0.001 for both), not by the cost of the perioperative care (*p* = 0.682).

**Table 2:** Clinicopathologic outcomes of patients who underwent gastrectomy for gastric cancer using either articulating laparoscopic instruments (ALI group) or a robotic surgical system (robotic group) for lymph node dissection.

Variables	Group		*p*
	ALI (n = 88)	Robotic (n = 96)	
Extent of LN dissection			0.760
< D2	56 (63.6)	59 (61.5)	
≥ D2	32 (36.4)	37 (38.5)	
Extent of gastrectomy			0.945
Distal gastrectomy	73 (83.0)	80 (83.3)	
Total gastrectomy	15 (17.0)	16 (16.7)	
Type of reconstruction			0.228
Billroth I	33 (37.5)	26 (27.1)	
Billroth II	38 (43.2)	53 (55.2)	
Roux-en-Y	17 (19.3)	17 (17.7)	
Operation time, min	168.6 ± 48.9	186.5 ± 58.6	0.026
Blood loss, mL	68.8 ± 61.1	61.1 ± 74.8	0.502
No. of collected LNs	34.0 ± 13.5	36.2 ± 14.4	0.289
No. of positive LNs	0.9 ± 2.3	1.3 ± 4.5	0.422
T classification			0.798
T1	65	72	
T2	4	7	
T3	12	10	
T4a	7	7	
N classification			0.167
N0	71	73	
N1	7	11	
N2	6	7	
N3a	4	1	
N3b	0	4	

Data are expressed as mean ± standard error or number (percentage).

ALI, articulating laparoscopic instrument; LN, lymph node.

**Table 3:** Postoperative outcomes and costs of patients who underwent gastrectomy for gastric cancer using either articulating laparoscopic instruments (ALI group) or a robotic surgical system (robotic group) for lymph node dissection.

Variables	Group		*p*
	ALI (n = 88)	Robotic (n = 96)	
Hospital stay, days	4.5 ± 1.7	4.1 ± 1.1	0.057
Time to passing gas, days	2.6 ± 0.7	2.7 ± 0.7	0.153
Early complications^a^			0.535
None	49	55	
C-D grade I	18	20	
C-D grade II	19	20	
C-D grade IIIa	2	0	
C-D grade IIIb	0	1	
Mortality^a^	0 (0.0)	0 (0.0)	NA
Total cost, USD	9822 ± 162	11,178 ± 202	< 0.001
Hospital stay cost, USD	813 ± 49	978 ± 50	0.020
Operation cost, USD	6303 ± 96	7546 ± 149	< 0.001
Perioperative care, USD	2705 ± 96	2654 ± 82	0.682

Data are expressed as mean ± standard error or number (percentage).

ALI, articulating laparoscopic instrument; C-D, Clavien-Dindo; NA, not applicable.

^a^In the first 30 days after surgery.

## Discussion

In this study, we compared the outcomes of patients who underwent MIS gastrectomy for gastric cancer using either ALIs or a robotic surgical system for lymphadenectomy. Our results revealed no significant differences in clinicopathologic outcomes between the two techniques except for a longer operation time in patients who underwent surgery using the robotic system.

One reason why we focused on laparoscopic surgery with ALIs versus surgery using a robotic surgical system in this study was to investigate whether “selectively using articulation function” affected the outcomes of patients who underwent MIS for gastric cancer. Of the two techniques, the robotic surgical system was introduced earlier for use in gastric cancer surgery compared to ALIs. Therefore, we have more accumulated expertise performing robotic gastrectomy for gastric cancer patients. In our experience, articulation, which is a key characteristic of robotic instruments, is not necessary for the entire duration of gastric cancer surgery. Specifically, the clinical usefulness of articulation is apparent during lymphadenectomy, but not during the reconstruction phase. Therefore, in clinical practice, many surgeons use laparoscopic procedures after completing lymphadenectomy during robotic gastrectomy, implying that the articulating function of robotic instruments does not benefit the reconstruction process^18^. However, articulation is of substantial benefit during some portions of the lymphadenectomy procedure. For example, when we perform dissections in LN stations no. 8a, 11p, and 12a, the hepatic artery or pancreas act as a major hindrance in retrieving the nodes, and an overly aggressive approach using conventional laparoscopic instruments (CLIs) for LNs located behind natural obstacles may result in injury to major vessels (e.g., hepatic artery, portal vein, splenic vessels) or the pancreas^32^. To overcome these obstacles in the supra-pancreatic area, we used instrument articulation provided by the robotic surgical system.

When retrieving LNs in these difficult areas, ALIs possess a clear advantage over CLIs. The end-effector part of ALIs resembles that of the robotic instrument, and ALIs provide a similar DOF as the robotic surgical system. These features allow ALIs to be able to handle tissues in the “deep” surgical field, where the target is so far from the trocars that two working instruments form a nearly parallel position. Currently, the cutting edges of each instrument cannot work properly when stiff laparoscopic instruments (i.e., CLIs) are used. However, articulating devices can successfully reach the target, providing triangular traction appropriate for dissection using both ALIs and robotic instruments.

Although ALIs considerably mimic the DOF of robotic instruments, achieving a specific angulation can be more challenging with ALIs compared to a robotic surgical system for several reasons. First, since the “control” part of ALI is composed of bulky structures (which are essential for achieving multi-DOF function), the surgeon’s wrist must bear the heavier weight of ALIs, compared to CLIs^33^. Second, as ALIs do not provide any axial rotation (which is provided in the robotic surgical system), surgeons must induce the rotation of the end-effector part of ALIs by pivoting their wrists, a technically challenging action. Finally, while the surgeon’s two fingers are sufficient to handle the master controllers suspended from the console cart during robotic surgery, the control part of ALIs must be adjusted by the surgeon’s wrist when manipulating the end-effector part of the ALI, potentially leading to fatigue in surgeons who utilize ALIs^33^.

For the above-mentioned reasons, the use of ALIs may result in some physical discomfort for surgeons. Nevertheless, ALIs can be a solution for situations where CLIs exhibit limitations. As shown by our results, robotic gastrectomy requires approximately 3 h to complete, but the articulating function is not necessary for every portion of the gastrectomy. Therefore, it is reasonable to use articulating instruments only during the portion of the procedure requiring articulation. One such strategy for accomplishing this is to perform laparoscopic gastrectomy using ALIs. Although a steep learning curve is necessary to become accustomed to using ALIs, it is worthwhile to invest the time and effort in learning to use these instruments so that they can be an option for procedures where articulation is critical. The clinical usefulness of ALIs is more immediately apparent compared to the usefulness of agents to avoid bleeding or adhesions-events that can be unpredictable or delayed and which surgeons invest considerable time and expense trying to avoid.

While this study is strengthened by its prospective design, one limitation is that our study design did not allow us to determine why the operation time was shorter in the ALI group compared to the robotic group. Possible explanations include the shorter time required during laparoscopic gastrectomy with ALIs for exchanging instruments or cleaning the lens of the scope. To acquire more insight into this and other important issues, we are planning a multinational study (NCT05550974) to investigate the types of procedures requiring ALIs, how ALIs affect the procedure, surgeon compliance with ALI usage, and the types of ALIs used during each portion of the surgery.

In conclusion, laparoscopic gastrectomy using ALIs was associated with comparable perioperative surgical outcomes and faster operation time compared to robotic gastrectomy in this prospective cohort study. Further comparisons with conventional laparoscopic surgery and external validation of these findings are necessary.

## Data Availability

The datasets used and/or analysed during the current study available from the corresponding author on reasonable request.
